# Microleakage in class II restorations of two bulk fill resin composites and a conventional nanohybrid resin composite: an in vitro study at 10,000 thermocycles

**DOI:** 10.1186/s12903-021-01942-0

**Published:** 2021-12-04

**Authors:** César F. Cayo-Rojas, Karen K. Hernández-Caba, Ana S. Aliaga-Mariñas, Marysela I. Ladera-Castañeda, Luis A. Cervantes-Ganoza

**Affiliations:** 1 School of Stomatology, Universidad Privada San Juan Bautista, Jose Antonio Lavalle Avenue No 302 – 304 (Ex Hacienda Villa); Chorrillos, Lima, Peru; 2 Faculty of Dentistry and Posgraduate School, Universidad Nacional Federico Villarreal, Lima, Peru; 3 Faculty of Stomatology, Universidad Inca Garcilaso de La Vega, Lima, Peru

**Keywords:** Monoblock resin, Composite resin, Marginal adaptation, Microleakage, Molar, Mono incremental resin, Silver nitrate, Thermal cycling, Tooth preparation

## Abstract

**Background:**

The contraction presented by resin composites causes an increase in stress at the tooth-resin interface, causing micro-gaps that allow microleakage. This study aims to evaluate the degree of in vitro marginal microleakage in class II restorations with two bulk fill resin composites compared to a conventional nanohybrid resin composite.

**Methods:**

The present study was an in vitro experimental design. A total of 30 standardized class II cavities were prepared in 15 human molars (mesially and distally). These cavities were later distributed in 3 groups according to the type of resin. Groups A and B were restored with bulk fill resin composites (Filtek—3 M/ESPE and Tetric N-Ceram—Ivoclar/Vivadent respectively) in a single increment of 4 mm. Group C was restored with the Filtek Z350 XT – 3 M/ESPE resin composite and two increments of 2 mm. Later, the restorations were subjected to 10,000 thermocycles between 5 °C to 55 °C and immersed in a silver nitrate solution (1 M for 24 h). The crowns were then sectioned mesiodistally and observed under the stereomicroscope to determine the degree of marginal microleakage at the occlusal and cervical areas. The results were analyzed with the Kruskal–Wallis and the Mann–Whitney U statistical tests.

**Results:**

There were no statistically significant differences regarding the degree of microleakage between the three types of resin composites in the occlusal and cervical areas (*p* > 0.05). Similarly, there were no significant differences after comparing each resin type in its occlusal and cervical area (*p* > 0.05).

**Conclusion:**

Filtek Bulk Fill and Tetric N-Ceram Bulk Fill resin composites showed no statistically significant differences with the conventional nanohybrid resin composite Filtek Z350XT at both occlusal and cervical areas.

## Background

Nowadays resin composites are the most employed dental restoration materials because of their good physicochemical properties and their excellent aesthetics [1]. Over time there has been an optimization of these properties and the restoration techniques in which they are employed. However, despite progress, polymerization shrinkage of resin composites remains a challenge [2].

Polymerization shrinkage generates stress that can damage the bond of the resin composites to the cavity walls, which produces microleakage allowing bacteria and fluids to move via the tooth-restoration interface [3]. This marginal microleakage has a negative influence on the longevity of dental restorations because it can produce recurrent caries, hypersensitivity, discolorations, and pulpal lesions, among others [4]. Microleakage remains a cause of failure in direct posterior restorations and is a factor to be considered in order to ensure the longevity of dental restorations [5].

The introduction of bulk fill resin composites has led to controversy over their use compared to incremental resin composites, based on its bulk application and the shorter time consumed in dental preparations. [6]. These bulk-fill resin composites make it possible to light-cure a 4 to 5 mm deep resin layer without prolonging the light exposure time or affecting the marginal adaptation of the restorative material [7]. In addition, they offer less polymerization shrinkage, good bond strength, and a high clinical effectiveness [8]. Moreover, they are a good alternative to be employed in non-cooperative patients [9].

The Filtek Bulk Fill resin composite contains two new methacrylate monomers: AUDMA (aromatic urethane dimethacrylate) and AFM (addition-fragmentation monomer), which help to reduce shrinkage stress while maintaining physical properties. Its presentation comes in semi-translucent tones that allow for a depth of polymerization superior to conventional resins. [10]. On the other hand, there have not been any changes regarding the polymerization initiation system in most of the bulk fill resin composites, except for the Tetric N-Ceram Bulk Fill, in which a new initiator called ivocerin, which has a higher reactivity compared to camphorquinone, was added. This initiator increases the polymerization depth to 4 mm and reduces the clinical working time. In addition, Tetric N-Ceram Bulk Fill has two pre-polymers and filler particles (isofillers) that reduce the shrinkage stress during polymerization [11, 12].

Several studies have evaluated the marginal microleakage of bulk fill resin composites, which show a varied composition among them [13–19]. And also, several studies have not been able to find statistically significant differences between bulk fill resin composites and the conventional ones [14–16]. Versluis et al. [17] concluded that the incremental technique applied to conventional resins increased the deformation of the restored tooth due to the incremental deformation of the preparation, causing a higher stress at the tooth-restoration interface than the stress caused by the bulk technique applied with bulk fill resin composites [18, 20].

In most studies there is no consistency in the in vitro experimental procedures regarding light intensity in the photoactivation unit, the amount of thermal cycles to accelerate the aging process of the resin composite, the number of increments that could lead to bubble accumulation and thus, the creation of microleakage at the resin-tooth interface that would influence the evaluation studies. In addition, it is known that greater the separation between the photoactivation unit and the resin composite, the lower the irradiance, and this could cause an inadequate activation of the monomers in the deeper section of the class II cavity [13–23].

On the other hand, numerous studies have evaluated the marginal sealing performance in class II restorations with a bulk fill composite resin type compared to a conventional nano-hybrid composite resin after applying artificial aging with 500, 1000, 1500 and 5000 thermal cycles. [14, 16, 21, 22]. Unlike these, in the present study it was decided to evaluate the microfiltration both in cervical and occlusal, but in three different resin composites, increasing the thermal cycles to 10,000. Therefore, the aim of this study was to evaluate the degree of in vitro marginal microleakage in class II restorations with two bulk fill resin composites compared to a conventional nanohybrid resin composite. The null hypothesis was that there is no statistically significant differences between restorations in class II cavities when comparing two bulk fill resin composites with a conventional resin composite.

## Methods

### Sample calculation and selection

15 healthy molars, extracted for orthodontic or prosthetic reasons, were collected from informed patients who agreed to voluntarily donate their teeth for research purposes, respecting the Declaration of Helsinki. The molars were extracted at the Adult Dental Clinic of the Inca Garcilaso de la Vega University. A total of 30 class II cavities were prepared. The sample size was 10 class II cavities for each resin composite group and was calculated based on the data obtained in a previous pilot study, where the ratio comparison formula was applied considering a P_1_ = 0.8, P_2_ = 0.2, α = 0.05 and a 1—β = 0.8. The teeth were randomly distributed in three groups (A, B and C) as follows:

- Group A: for Filtek Bulk Fill, shade A2 (3 M ESPE, St. Paul, MN, USA).
- Group B: for Tetric N-Ceram Bulk Fill, shade A2 (Ivoclar Vivadent AG, Schaan, Liechtenstein).
- Group C: for Filtek Z350 XT, shade A2 (3 M ESPE, St. Paul, MN, USA).

### Tooth preparation

**Fig. 1:**
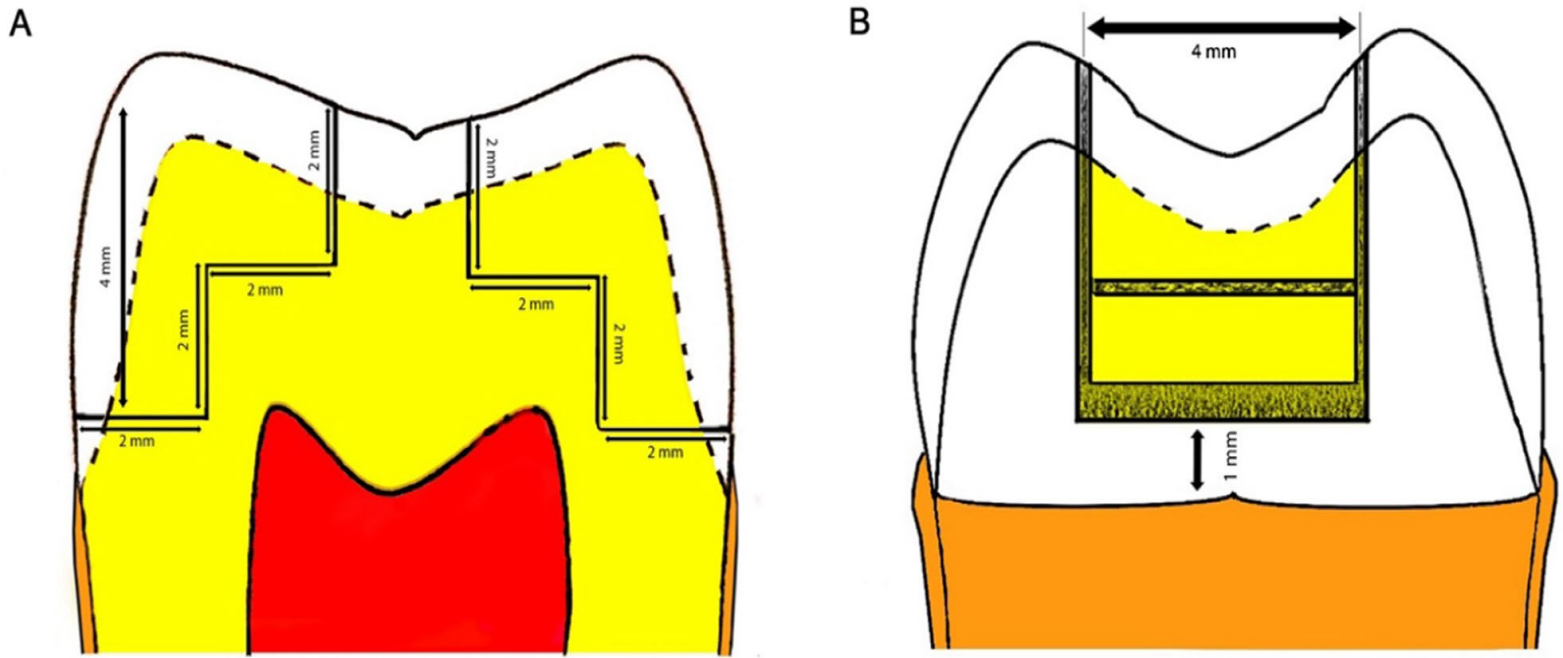
Class II cavity preparation. **a** Crown sectioned lengthwise (mesiodistal direction). **b** Proximal view

The teeth were cleaned and immersed in a 1% chloramine-T solution for one week to be disinfected. After this time, they were placed in distilled water at 4 °C with refills every 7 days. The teeth were kept no longer than 6 months after their extraction and were conditioned in distilled water at 23 ± 2 °C for 24 h prior to cavity preparations. All cavity preparations and restorations were performed by the same operator, with a high-speed handpiece (NSK Pana-Max PAX-TU M4, Tochigi, Japan) with cooling, using a cylindrical diamond bur No. 1092 (Microdont, Sao Paulo, Brazil), which was changed every 5 cavity preparations. Two class II cavities were prepared in each tooth with a 90° cavosurface angle, standardized for both the occlusal box and the proximal box (Fig. 1). In addition, the depth of the proximal cavity was made 1 mm above the cement-enamel junction. All dimensions were measured with a WHO periodontal probe (Hu Friedy, Chicago, USA) with a standard deviation of ± 0.2 mm.

### Cavity conditioning

The cavities in all three groups (A, B and C) were etched with 35% phosphoric acid gel (Ultra-Etch, Ivoclar Vivadent AG, Schaan, Liechtenstein) for 20 s, then rinsed for 5 s and partially dried with pieces of gauze until the dentine was moist. A layer of fifth generation adhesive (Adper Single Bond-2, 3 M ESPE, St. Paul, MN, USA) was then applied with a microbrush to all surfaces of the cavity, followed by a gentle stream of air over the liquid for about 5 s until the solvent evaporates completely [24, 25]. Then, a LED light-curing unit (Bluephase N, Ivoclar Vivadent AG, Schaan, Liechtenstein) was used at a light intensity of 1200 mW/cm^2^ for 10 s. A circumferential metallic matrix (Automatrix MT, Dentsply, Milford, DE, USA) was adjusted around each cavity for an adequate conformation of the restoration walls.

### Cavity restorations

Group A and B cavities were restored using a single 4 mm block filler and light cured for 10 s from occlusal to a light intensity of 1200 mW/cm2. The metal matrix was then removed to light cure from buccal and lingual for an additional 10 s. The cavities in group C were incrementally restored with two 2 mm layers and light cured consecutively for 10 s with the same LED light-curing unit and equal light intensity. This intensity was checked with a radiometer (Bluephase Meter II, Ivoclar Vivadent AG, Schaan, Liechtenstein).

Immediately after completion of the tooth restoration procedure, the teeth were placed in distilled water at 37 ± 2 °C using an incubator for 24 h and then finished with abrasive discs (Sof-lex, 3 M ESPE, St. Paul, MN, USA).

### Thermocycling, preparation, and immersion of teeth in dye

The filled teeth were subjected to 10,000 thermocycles between 5 °C and 55 °C, with 30 s of exposure in each bath and 10 s of transfer time between baths. At the end of this process, the teeth surfaces were covered with two coats of nail varnish up to 1 mm before the restoration limits. The dental apexes were sealed with self-curing acrylic (Vitacryl, Vitalloy, Lima, Peru) to prevent penetration of the dye through the apex. The samples were then immersed in a 1 M silver nitrate solution for 24 h without exposure to light. At the end of this time, the samples were washed with abundant water for 5 min and then placed in a photodevelopment solution under fluorescent light for 8 h. Finally, they were rinsed and checked to ensure that dye had not entered through the apex.

### Sample sectioning for observation under stereomicroscope, observer calibration, and score.

**Fig. 2:**
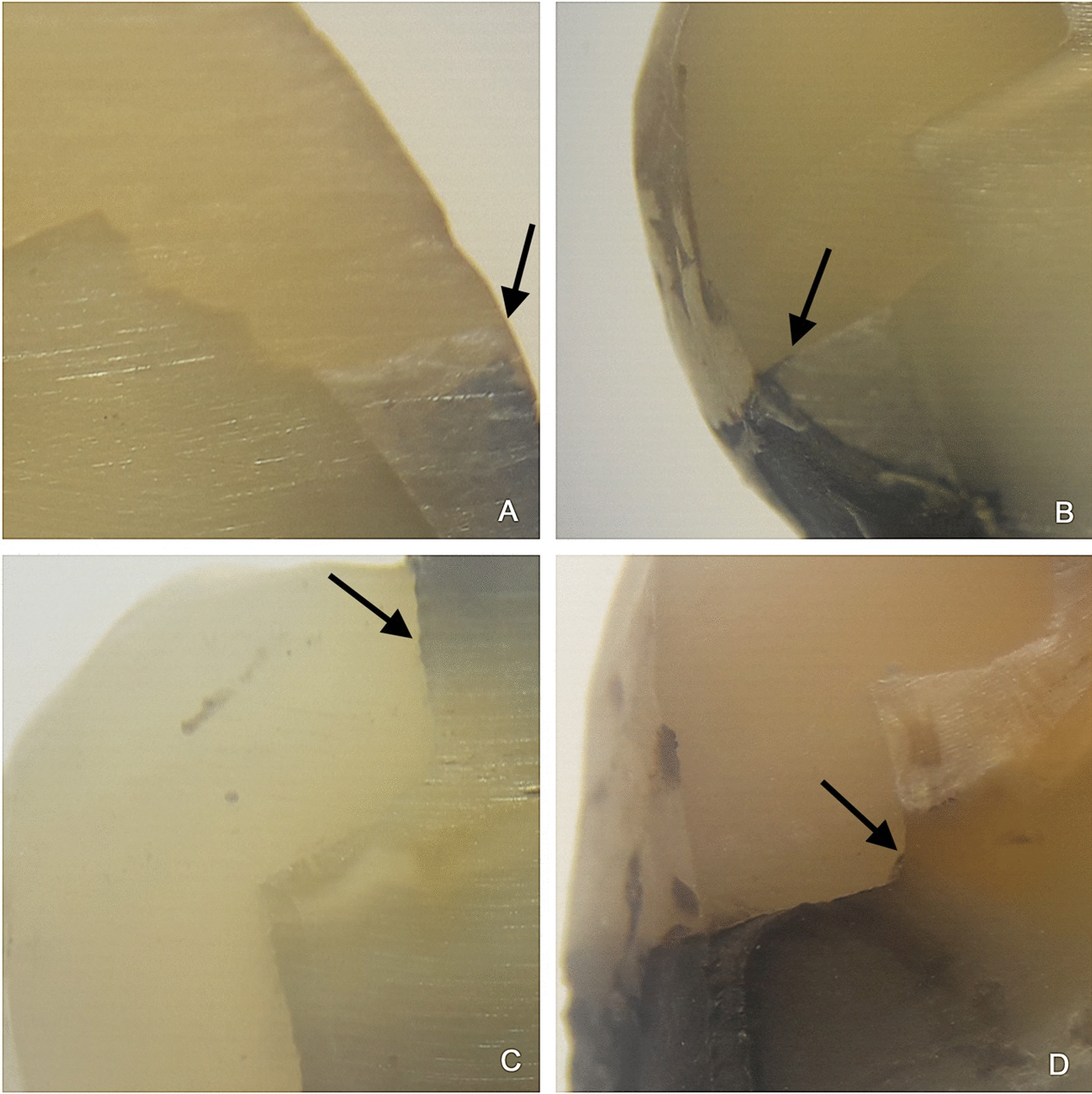
Marginal microleakage of bulk fill resin composites. **a** Score 0 (No penetration), **b** Score 1 (Penetration into the enamel of cavity wall), **c** Score 2 (Penetration into the dentin of cavity wall without including the pulpal wall of the cavity), **d** Score 3 (Penetration including the pulpal wall of the cavity)

The roots of the teeth were cross sectioned 3 mm below the cement-enamel junction. Immediately after, the crowns were sectioned lengthwise (in mesiodistal direction) with double-sided diamond discs with a thickness of 0.20 mm placed in a low-speed handpiece (Strong 210, Saeshin, Korea), and abundant irrigation. The sectioned surfaces were polished with silicon carbide papers under a stream of water for 2 min and later dried for stereomicroscope observation (Leica EZ4, Wetzlar, Germany) at × 16 magnification to register the degree of marginal microleakage. Stereomicroscopic reading of samples was performed by an expert in histology. In addition, an intra-examiner (0.90; CI: 0.65—1.00) and inter-examiner (0.78; CI: 0.48—1.00) calibration was performed using the Kappa index, and these results were acceptable. A double-blind procedure was applied. Both the statistician and the expert who performed the readings under the stereomicroscope were unaware of the group assignment. In order to measure the silver nitrate penetration through the cavity walls, we employed the scoring system provided by *International Organization for Standardization* PD ISO/TS 1145:2015 [26]. (Fig. 2).

### Statistical analysis

The statistical analysis was performed with SPSS 25.0. Since the data did not show a normal distribution, the Kruskal–Wallis test was employed to compare the degree of microleakage in the three types of resins both in the occlusal and cervical areas, and the microleakage between the occlusal and cervical area of each group was compared by means of the Mann–Whitney U test. The differences were considered statistically significant for *p* < 0.05.

## Results

In the occlusal area, the microfiltration score for the Filtek Bulk Fill resin composite was 0 in 80% of the cases whereas the microleakage score for the Tetric N-Ceram Bulk Fill was 1 in 70% of the cases. The microleakage of the Filtek Z350 XT resin composite had a similar distribution among the scores 0, 1 and 2.

In the cervical area, the microleakage score for the Filtek Bulk Fill resin composite was 0 in 50% of the cases whereas the score for the Tetric N-Ceram Bulk Fill was 1 in 50% of the cases, and the score for the Filtek Z350 XT resin composite was 1 in 60% of the cases. (Table 1).

**Table 1:** Marginal microfiltration score in the occlusal and cervical area according to each type of resin evaluated

Resin composites	Area	Score 0		Score 1		Score 2		Score 3		Total	
		f	%	f	%	f	%	f	%	N	%
Filtek Bulk Fill	Occlusal	8	80%	1	10%	0	0%	1	10%	10	100%
	Cervical	5	50%	3	30%	2	20%	0	0%	10	100%
Tetric N-Ceram Bulk Fill	Occlusal	3	30%	7	70%	0	0%	0	0%	10	100%
	Cervical	1	10%	5	50%	3	30%	1	10%	10	100%
Filtek Z350 XT	Occlusal	4	40%	3	30%	3	30%	0	0%	10	100%
	Cervical	2	20%	6	60%	1	10%	1	10%	10	100%

f: absolute frequency; n: sample size

When comparing the degree of marginal microleakage in the three types of resin composites, we did not obtain statistically significant differences in the occlusal (p = 0.149) and cervical (p = 0.180) area. Additionally, the Filtek Bulk Fill resin composite showed a minor degree of microleakage in both the occlusal (M_e_ = 0) and cervical (M_e_ = 0.50) area, which means that in most cases it did not show microleakage in any of these two areas. (Table 2).

**Table 2:** Central tendency measurement of the degree of marginal microleakage in resin composites according to the treatment area

Resin composites	Area	Median	(IQR)	Range	p-value*
Filtek Bulk Fill	Occlusal	0		3	0.149
Tetric N-ceram bulk fill		1	(1)	1	
Filtek Z350 XT		1	(2)	2	
Filtek bulk fill	Cervical	0.50	(1)	2	0.180
Tetric N-ceram bulk fill		1	(1)	3	
Filtek Z350 XT		1	(1)	3	

IQR: Interquartile Range; *Based on the non-parametric Kruskal–Wallis test for individual samples; * Statistically significant difference *p* < 0.05

When analyzing the degree of marginal microleakage between the occlusal and cervical areas of each resin studied, we did not obtain significant differences for any of the three: Filtek Bulk Fill (p = 0.315), Tetric N-Ceram Bulk Fill (p = 0.075) and Filtek Z350 XT (p = 0.684) respectively. As can be seen, the Tetric N-Ceram Bulk Fill resin composite showed more differences in the degree of microleakage at both the occlusal and cervical areas. (Table 3).

**Table 3:** Central tendency measurement of the degree of marginal microleakage in each treatment area according to the type of resin composite applied

Resin composites	Area	Median	(IQR)	Range	p-value*
Filtek Bulk Fill	Occlusal	0	(0)	3	0.315
	Cervical	0.50	(1)	2	
Tetric N-Ceram Bulk Fill	Occlusal	1	(1)	1	0.075
	Cervical	1	(1)	3	
Filtek Z350 XT	Occlusal	1	(2)	2	0.684
	Cervical	1	(1)	3	

IQR: Interquartile Range; *Based on the non-parametric Mann–Whitney U test for individual samples; *Statistically significant difference *p* < 0.05

## Discussion

This study evaluated the degree of marginal microleakage of class II restorations in two bulk fill resin composites and an incremental nanohybrid resin composite. We did not obtain statistically significant differences in the occlusal and cervical areas when comparing the three groups of resin composites. Moreover, we did not find significant differences when analyzing the microleakage in the occlusal and cervical area for each type of resin composite. Therefore, the null hypothesis was accepted.

Tests performed in this study were limited to the evaluation of the microleakage under the stereomicroscope. We did not use of the scanning electron microscope (SEM) with energy-dispersive X-ray spectroscopy (EDS) because the purpose was not to quantify the amount of silver ions in the resin-tooth interface but to determine the degree of penetration of silver nitrate through the interface. Therefore, to accomplish this goal we decided to evaluate the marginal microleakage of the resin composites under the stereomicroscope as numerous studies supported this type of evaluation [14, 16, 21, 22, 24, 27].

In order to simulate the temperature variation that occurs in the oral cavity equivalent to a year of clinical aging [22, 23, 28–31], 10,000 thermocycles were applied, which is a superior quantity regarding the 500, 1000, 1500 and 5000 thermocycles employed in the majority of the studies aiming at analyzing the best performance of marginal sealing in class II restorations with a type of bulk fill resin composite versus a conventional one [14, 16, 21, 22]. This research used 1 M silver nitrate as dye [14, 32] as it is one of the most employed dyes in microleakage and nanoleakage studies due to the fact that silver ions have a good diffusion capacity across the resin-tooth interface and also absorb light, reducing silver diamine ions with a diameter of 0.059 nm to metallic silver grains, which makes easier the stereomicroscope observation when compared to methylene blue [27, 33].

There is evidence that the cavity design has an influence on the stress caused by the shrinkage of the resin composite during polymerization which may bias the results [34]. However, there is no gold standard rule on the measurements for cavitary preparations to carry out this kind of studies. In this study, in order to reduce bias, not only the cavities were standardized but also the acid etching procedure, the light curing mode and the use of adhesive for all samples.

The three types of resin composites evaluated in this study presented a low degree of marginal microleakage in most cases. Nevertheless, the Filtek Bulk Fill resin composite showed a lower degree of microleakage in both the occlusal and cervical areas when compared to the Tetric N-Ceram Bulk Fill resin composite and the conventional resin composite Filtek Z350 XT. Even though this outcome was not statistically significant, the results were similar to those from numerous authors [6, 14–16, 21, 35]. This slight difference is probably supported by the fact that the Filtek Bulk Fill resin composite has two new methacrylate monomers, AUDMA and AFM, which helped to reduce the shrinkage stress while keeping the physical properties, leading to a reduction in the formation of microgaps at the resin-tooth interface. This fact was more noticeable in the occlusal area than in the cervical area. [10]. The slight dissimilarity between the two areas is probably due to the metallic matrix employed in the proximal box, which absorbed the photons available for photoactivation. A possible consequence of this is the lack of polymerization in the deep zone of the restoration [36].

The Tetric N-Ceram Bulk Fill resin composite has camphorquinone as the main photoactivator, which absorbs a blue wavelength from 420 to 495 nm, with 468 nm as its maximum absorption peak. In addition, it has alternative photoinitiators, such as acylphosphine oxide (Lucerin TPO) and dibenzoyl germanium derivatives (Ivocerin), which absorbs a wavelength from 370 to 460 nm, with 408 nm as its absorption peak [11, 12]. It has been proven that with these alternative photoinitiators, which are activated with LED *poliwave* (Bluephase N), it is possible to get an optimum polymerization until 2.5 mm of depth. Nevertheless, the efficacy of this activation decreases at greater depth because violet light cannot reach the deeper zone of the restoration due to the condensation of molecules at a surface level [37]. This issue probably had an influence in the fact that Tetric N-Ceram Bulk Fill resin composite presents more microleakage in the cervical area than the occlusal area, although not significantly.

The Filtek Z350 XT resin composite presented greater microleakage than the Filtek Bulk Fill resin composite but not significantly, probably because placing more than 1 resin composite increment can cause microbubbles between layers, which lead to microgaps in the resin-tooth interface allowing for increased penetration of the silver nitrate [38].

There are some dissenting studies [32, 39] with the results obtained in this research. Other authors have reported significant differences of marginal microleakage in class II restorations between the occlusal and cervical areas when comparing bulk fill resin composites with conventional nanohybrid resin composites. It is probable that this discrepancy is due to the fact that the authors made the cervical margins of the class II cavities below the cement-enamel junction. However, the present study prepared the proximal box over this junction since it has been demonstrated [16] that the cervical microleakage at 1 mm below the cement-enamel junction is significantly higher than at 1 mm above this junction. The reason is that the adhesion of acid etching with enamel is better than with cement due to the fact that enamel has a higher inorganic composition (95%) and less moisture. On the other hand, cement adhesion is weaker than mantle dentin adhesion because the latter has thicker collagen fibers (0.1 – 0.2 um) and a higher amount of hydroxyapatite with a 20% to 24% of difference between these tissues [40–42].

A limitation of the present study was that the experiments were performed on in vitro teeth with artificial aging and we did not quantify the amount of silver ions present in the microgaps of the resin-tooth interface since the scanning electron microscope with energy-dispersive X-ray spectroscopy were not employed. Additionally, there were not comparisons between the microleakage at different thermal cycles.

The results obtained in this study should be taken with caution due to the existence of studies indicating that in vivo results are not always similar to in vitro results. However, due to the little clinical evidence comparing the three resin composites described in this study, it is necessary to recommend randomized clinical trials analyzing the microleakage in class II cavities of bulk fill resin composites and incremental nanohybrid resin composites of different commercial brands, which should be studied with scanning or transmission electron microscopy and with the use of 1 M silver nitrate. In this way, it would be possible to quantify the amount of silver ions in the microgaps of the resin-tooth interface by EDS.

## Conclusions

Considering the limitations of the present in vitro study, it can be concluded that Filtek Bulk Fill resin composite and Tetric N-Ceram Bulk Fill resin composite did not present statistically significant differences compared to conventional nanohybrid Filtek Z350 XT resin composite in the area occlusal and cervical. However, it is advisable to use Bulk fill resin composites in class II restorations, as their single-layer placement may reduce the clinical working time.

## Data Availability

Data are available upon request at cesarcayorojas@gmail.com.

## Abbreviations

AFM: Addition-fragmentation monomer
AUDMA: Aromatic urethane dimethacrylate
EDS: Energy dispersive X-ray spectroscopy
LED: Light-emitting diode
SEM: Scanning electron microscope
TPO: Trimethylbenzoyldiphenylphosphine oxide
WHO: World Health Organization
